# Thymopoietin-*α*, -*β*, and -*γ* Isoforms Increased Expression in Cervical Cancer Cells

**DOI:** 10.1155/cjid/1668482

**Published:** 2025-04-09

**Authors:** Víctor Huerta-Padilla, Daniel Marrero-Rodríguez, Keiko Taniguchi-Ponciano, Ariana E. López, Fernando Candanedo-González, Emmanuel Salcedo, Alejandra Valdivia-Flores, Miriam Rodriguez-Esquivel, Laura Gómez Virgilio, Ricardo López-Romero, Maria de Jesus Nambo-Lucio, Sergio E. Meza-Toledo, Cindy Bandala, Marco A. Meraz, Mauricio Salcedo

**Affiliations:** ^1^Oncology Genomics Biomedical Research Unit, Gynecology Pediatrics Hospital 3A, North Unity OOAD, Mexican Institute of Social Security, Mexico City, Mexico; ^2^Departamento de Bioquimica, Laboratorio de Quimioterapia Experimental, Escuela Nacional de Ciencias Biológicas, Instituto Politecnico Nacional, Mexico City, Mexico; ^3^Endocrine Diseases Research Unit, Specialties Hospital, National Medical Center SXXI, Mexican Institute of Social Security, Mexico City, Mexico; ^4^Anatomo-Pathology Service, Oncology Hospital, National Medical Center SXXI, Mexican Institute of Social Security, Mexico City, Mexico; ^5^Escuela Nacional de Ciencias Biologicas, Instituto Politecnico Nacional, Mexico City, Mexico; ^6^Centro de Investigación y de Estudios Avanzados, Molecular Biomedicine Department, Instituto Politecnico Nacional, Mexico City, Mexico; ^7^Londres Clinic, Angels Hospital, Ciudad de Mexico, Mexico; ^8^Departamento de Medicina Traslacional aplicada a Neurociencias, Escuela Superior de Medicina, Instituto Politecnico Nacional, Mexico City, Mexico

**Keywords:** cervical cancer, expression, HPV, isoforms, MCM2, thymopoietin

## Abstract

Cervical cancer (CC) is a public health concern related to the human papillomavirus (HPV) persistent infection. Minichromosome maintenance 2 (MCM2) has been postulated as a surrogate marker for HPV infection. Thymopoietin (TMPO) is a nuclear protein regulated by E2F such as MCM2 or p16. TMPO can give rise to six different isoforms. Herein, both the mRNA and protein levels of TMPO isoforms were analyzed in cervical cells. TMPO expression was selected and analyzed through in silico in several databases from the healthy cervix and cervical lesions. TMPO RNA expression was evaluated in cervical samples and cell lines by RT-PCR and protein expression by Western-blot and immunohistochemistry assays. TMPO and MCM2 immunostaining were evaluated in cervical smears. The clinical-pathological correlation analysis was performed using Kruskal–Wallis or *Χ*^2^ tests. TMPO is overexpressed in 74% of CC cells and all CC cell lines. Moreover, negative immunostaining was observed in normal cervical tissue, compared to strong expression for cervical lesions. Interestingly, TMPO-α, -β, -δ, -ε, and -γ are expressed in all cervical cells and tissues, but a differential expression for α, -β, and -γ isoforms among the cervical cells was observed as overexpressed when HPV is present. Also, the immunostaining of both MCM2 and TMPO was quite similar, but TMPO expression was more sensitive and specific than MCM2 protein. The present study has revealed that TMPO protein expression could be a potential molecular marker for cervical transformed cells, highlighting the TMPO-α, -β, and -γ isoforms as a promising molecular marker of HPV infection.

## 1. Introduction

Cervical Cancer (CC) and its precursor high-grade squamous intraepithelial lesions have been established as important health concerns, and several modifiable risk factors are associated with these lesions. Human papillomavirus (HPV) infection acquired in most cases through sexual intercourse, is considered as the main etiological factor [1].

In these lesions, the high-risk-HPV *E6* and *E7* oncogenes are expressed in the epithelial cells resulting in “apparent dysregulation” of cell cycle control via p53 and pRB interference [2]. It is widely known that the E7 protein triggers p16 expression via E2F-pRb complex disruption [3]. Overexpression of the thymopoietin (TMPO) gene (also known as lamina-associated polypeptide 2 or LAP2) was also described in an E2F- and p53-dependent manner due to the presence of E6/E7 oncoproteins [4].

*TMPO* is cytogenetically located in the 12q23.1 region, composed of ∼35 kb of genomic DNA, with eight exons, coding nuclear proteins that participate in nuclear envelope architecture, among other functions. *TMPO* can give rise to six different isoforms generated by the splicing mechanism: *α*, *β*, *γ*, *δ*, *ε*, and *ζ*. These isoforms share a common N-terminal domain, and only the *α*- and *ζ*-isoforms lack the transmembrane domains, rendering these isoforms nucleoplasmic, while the remaining are anchored to the inner nuclear membrane [5–7]. Moreover, the minichromosome maintenance (MCM) proteins are a family of six structurally related proteins minichromosome maintenance 2 (MCM2)–7 (cytogenetically located at 3q21 chromosome region), and these have a pivotal role in many biological processes, for example, they are required for the cellular events such as transcription, proliferation, expansion, and DNA replication [8]. In addition, there is wide evidence that CC frequently presents chromosomal imbalance at chromosome region 3q21 indicating tha*t MCM is* an important key in the development of this type of cancer [9–11]. In this sense, the strong expression of MCM2 protein in HSIL may aid as a concatenated screening tool in detecting precancerous cervical lesions [12].

The goal of the current study was to analyze the different TMPO isoform expressions in different cervical lesion statuses; furthermore, the dual expression of TMPO and MCM2 were also evaluated in cervical smears.

## 2. Materials and Methods

### 2.1. Data Mining of Gene Expression

Expression libraries were collected from the Cancer Genome Anatomy Project and SAGEmap in the National Center for Biotechnology. For this, two HSIL libraries with 173,534 and 171,008 tags each were analyzed [13]. For normal (or healthy) cervical epithelium, three different libraries with 30,418 tags [14], 165,624 tags, and 181,224 tags were used [13]. Furthermore, eight normal (N) and 57 CC experiments by using the Affymetrix Human Genome U133 Plus 2.0 Gene Chip were downloaded and analyzed with the Partek Genomics Suite. These libraries were downloaded from the Gene Expression Omnibus (GEO) and Gene Expression Atlas (GSE2109) [15–17].

### 2.2. Cervical Samples Collection and Ethic

The current study was a cross-sectional, observational, and analytical of consecutive nonrandomized cases during the period of 2013. The ethics and scientific committee approved this study (Instituto Mexicano del Seguro Social [IMSS], CNIC2013-785-089).

Forty cervical biopsy samples were taken from consecutive cases (ages +18 years) affected with low-grade squamous intraepithelial lesions or LSIL (*n* = 9), HSIL (*n* = 7), or CC (*n* = 16 squamous cell carcinomas and *n* = 4 adenocarcinomas). These were collected by the oncologist from the Oncology Department, Hospital General de Mexico, at Mexico City after signed informed consent.

After this procedure, the patients received specific treatment such as electrosurgery for the SIL patients or chemo- or radiotherapy for the cancer patients [18]. The biopsies were cut into two fragments, one was fixed in buffered formalin for 12 h and processed in the Pathology Department; the histopathological status was confirmed by the pathologist. Another fragment was deposited in a microtube for DNA and RNA extraction according to manufacturer conditions.

For avoiding any bias of the result as contamination by conjunctive cells, only samples harboring more than 70% of the altered cells (neoplastic or transformed) were used for the experiments.

For normal patients (tissue control), 40 cervical samples were swabbed, smeared on a glass slide, and fixed by the gynecologist. The slides were stored in nonhumid conditions at 4°C until use. The pathologist confirmed the LSIL, HSIL, or normal status. Detailed clinicopathological information was obtained from the patient's records. Again, all the patients recruited signed informed consent before taking any sample according to protocol.

### 2.3. Culture Cell Lines

In the present work, three CC-derived cell lines were used. HeLa cells are an epithelial adenocarcinoma cell line harboring around HPV18 50 copies/cell with *p53* and *pRB* wildtype. SiHa cells are a squamous carcinoma cell line harboring HPV16 2 copies/cell also with *p53* and *pRB* wildtype; while the C33A cell line is an epithelial carcinoma cell line HPV-negative but *p53* and *pRB* mutated. These cell lines were grown on p-100 cell culture boxes with Dulbecco's modified eagle medium supplemented with 10% v/v of fetal bovine serum (FBS) and 1% penicillin/streptomycin. The cell lines were incubated under conditions of 37°C, 85% relative humidity, and 5% CO_2_ until reaching a confluence of 90% on the plate.

### 2.4. RNA Extraction and Reverse Transcription Reaction

The tissue samples and cells were homogenized in QIAzol Lysis Reagent and then incubated at room temperature (RT) for 5 min, and chloroform was added.

In brief, the total RNA was extracted from the aqueous phases using the RNAeasy tissue mini kit (Qiagen Inc. California, USA) according to the manufacturer's indications. RNA concentration was measured in a NanoDrop ND-1000 spectrophotometer (Thermo Scientific, Delaware, USA), and the RNA integrity was checked in 1.5% agarose gel. Immediately, 1 *μ*g of total RNA was retrotranscribed using the SuperScript VILO Master Mix (Applied Biosystems, California, USA), according to the manufacturer protocols.

### 2.5. DNA Extraction and HPV Detection

In parallel, DNA was extracted from the RNA extraction procedure's phenol phase, following 0.1 M sodium citrate in 10% ethanol, pH 8.5, washes (2X), and finally, the pellet was air-dried and DNA was resuspended in nuclease-free water.

HPV detection was performed by using oligos GP5+/GP6+ 20 pmol of each primer [19] and 100 ng DNA for 40 cycles of 94°C for 30 s and 55°C for 1.5 min, followed by 72°C for 1.5 min PCR conditions. The amplicons obtained were resolved in 2% agarose gel ethidium bromide-stained. For the PCR experiments, HeLa DNA was used as positive control; and a semiquantitative intensity analysis of the PCR products was finally done.

### 2.6. Analysis of the TMPO Isoform Transcripts by RT-PCR Endpoint in Cervical Tissue and Cell Lines

Specific primers for splicing-generated isoforms were in-house designed. PCR was carried out using the following primers 5′-TTC TTC CAG GGA GGC AAC ACA GAT-3′ and 5′-ATG GTA TGG GCA GCC ATC TTC ACT-3′ for TMPO-*α* with a product of 363 base pairs (bps) and 5′-AGA GAA CCA CTA AAG GGC AGA GCA-3′ and 5′-TTT GAT TGG TCT GCG GCA ACT AGC-3′ for the remaining 4 isoforms: TMPO-β = 510 bp, TMPO-ε = 390 bp, TMPO-δ = 294 bp, and TMPO-γ = 183 bp, except the TMPO-ζ isoform with conditions of 94°C for 7 min, 94°C for 45 s, 60°C for 45 s, 72°C for 45 s (35 cycles), and 72°C for 7 min.

RPS18 primers were used for housekeeping control according to a previous report [20]. PCR products were resolved in 2% agarose gel ethidium bromide-stained. To find differences in expression, again the intensity of each band was analyzed.

For qRT-PCR assays, all reagents were purchased from Applied Biosystems, California, USA, and by using 1 *μ*L of each TaqMan probe, 500 ng of cDNA was extracted in a 20 *μ*L final volume. In these cases, the human large ribosomal protein (RLPO) gene was used as the internal control, all reactions were performed in triplicate in the StepOne thermal cycler (Applied Biosystems), and finally, the relative expression by using the 2^−ΔΔCt^ equation was calculated.

### 2.7. MCM2 and TMPO Immunostaining Analysis of Cervical Tissue and Cervical Smear

A tissue microarray (TMA) including the 40 cases was constructed when the pathologist found the selected areas of the invasive tumor.

Also, a Pap stain was performed for the cervical smears and was reviewed by a pathologist to confirm the diagnosis.

Core samples for TMA were taken using 0.6 mm^2^ blunt-tip needles using a Tissue Microarrayer (Chemi-Con Co., Massachusetts, USA). To provide a sufficiently representative sample, each one was represented with the 2-fold redundancy. TMA slide sections (3 *μ*m) were used for the immunostaining assays using a streptavidin-biotin complex peroxidase method (Dako, Glostrup, Denmark).

For antigen retrieval, the TMA sections and cervical smear slides were treated in a pressure-cooked apparatus (15 min of maximum pressure) with Trilogy buffer. Endogenous peroxidase activity was inhibited with H_2_O_2_ in methanol solution.

In every case, the antigen detection was performed separately with the monoclonal antibody (mAB) anti-TMPO (L3414, Sigma-Aldrich, USA) and the mAB anti-MCM2 (Sigma-Aldrich, USA) for each one of the slides. These conditions were performed overnight at 4°C in a humidity chamber in a bovine serum albumin (BSA)–phosphate-buffered saline (PBS) buffer. Immunoreaction was developed with a peroxidase substrate solution (3,3-diaminobenzidine tetrahydrochloride, H_2_O_2_ in PBS), and finally counterstained with hematoxylin. Always positive and negative biological controls were used for each antibody reaction, and a technical negative control was replaced by the primary antibody with BSA solution, as described previously [20]. All experiments and analyses were performed at two independent times. Three independent observers performed immunostaining assessment at independent times by light microscopy at 20x magnification with a pathologist monitoring. The results were evaluated as negative, weak positive, moderate, and strong positive staining.

### 2.8. TMPO Expression in Cell Lines

HeLa, SiHa, and C33A cell lines' protein extracts were used for western-blot assays. The cells were centrifugated and resuspended in RIPA lysis buffer (1% Nonidet P-40, 0.5% sodium deoxycholate, and 0.1% SDS in IX PBS), protease inhibitors were added extemporaneously (0.1 m/mL PMSF, aprotinin, 0.18 mg/mL sodium orthovanadate), incubated for 30 min, and homogenized in a vortex. They were cold incubated for 30 min and centrifuged to discard the cellular waste. One aliquot of the supernatant collected was used to determine the protein concentration by using the BCA Protein Assay (Thermo Scientific #23225), while another aliquot was used for protein electrophoresis. For this, 1 *μ*g/*μ*L of proteins were denatured by boiling them in *β*-mercaptoethanol solution and consequently resolved in a 15% polyacrylamide/SDS gel electrophoresis in tris-glycine solution. This was performed with the Bio-Rad “mini-protean” system at 100 V for 1 h. The gels were equilibrated for subsequent transfer to a nitrocellulose membrane by wet transfer with the Bio-Rad mini trans-blot system in 25 mM tris/19 mm glycine/20% methanol pH8.4 transfer buffer at 0.5 A.

For immunodetection, the blot membranes were blocked with 5% milk powder and 0.05% Tween-20 in TBS pH 7.4 buffer and incubated with the mAb anti-human TMPO (cat. L3414, Sigma-Aldrich, USA), and then the color development was observed using the HRP chemiluminescence system with the Millipore “Immobilon Western” kit (Cat. no. WBKLS0500). The chemiluminescent-exposed membrane was developed using the Bio-Rad ChemiDoc system. The analysis was performed by densitometry using the ImageJ program.

### 2.9. TMPO Immunodetection Analysis

HeLa, SiHa, and C33A cell lines were used for the immunofluorescence (IF) assay. For this, the cells were incubated in 4% paraformaldehyde-fixed solution (PBS, pH 7.4) for 30 min. Then, the retrieval antigen was obtained using a 0.2% PBS/Triton solution in 30 min at RT.

A solution of PBS/Triton/albumin for 60 min to block nonspecific reactions was used after the mAB anti-TMPO (Sigma-Aldrich, USA) or mAB anti-human tubulin (Abcam, cat. Ab4074) with a 1:500 dilution in PBS/Triton/albumin for each antibody was incubated overnight at 4°C. After the cells were washed with PBS/Triton solution and incubated with the secondary antibodies (1:500 dilution), Goat anti-mouse Alexa 488 (Invitrogen, cat: A11029) was used to label the mAB anti-TMPO or the goat anti-rabbit secondary antibody Alexa 594 (Invitrogen, cat: A11037) was used to label the anti-*α*-tubulin antibody. This preparation was left incubating in the dark for 60 min at RT. Subsequently, the preparation was washed with a PBS/Triton solution, counterstained with DAPI stain (10 mg/mL), and finally mounted.

### 2.10. Statistical Analysis

The *X*^2^ test with the Fisher exact test was performed by the clinical and pathological correlation analysis. All *p* values were considered significant at *p* ≤ 0.05 and represented a two-tailed test. The parameters were dichotomized as positive or negative expression and the presence or absence of each isoform. Statistical analysis was performed using SPSS V21 statistical software.

## 3. Results

### 3.1. Clinical Data and TMPO Expression Detection

Analysis of the clinical-pathological variables showed that the known risk factors, such as age, smoking, the use of hormonal contraceptives, and alcohol intake, have no correlation with the TMPO expression. In contrast, a positive correlation was observed with HPV infection associated with colposcopic and histologic diagnoses with LSIL, HSIL, and CC (see Table 1).

The patients were 40.4 ± 15.11 years of age (range: 18–74 years). The data show that all CC samples (100%) harbored HPV infection, while HSIL and LSIL samples were 92.9% and 85% HPV-positive, respectively. The most frequent HPV types were HPV16, −18, −58, and −33 (data not shown).

### 3.2. Data Mining for TMPO Expression

**Figure 1:**
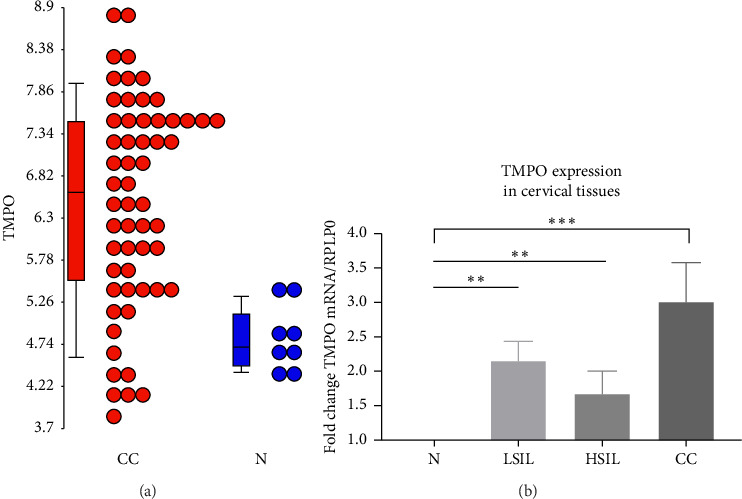
TMPO relative expression at mRNA level. (a) The in silico TMPO expression dot plot at the mRNA level. Blue dots represent TMPO expression levels in normal tissues, while the red dots are for CC samples. (b) The TMPO increased expression in precursor lesions (LSIL or HSIL) and CC compared to normal tissue (*p* ≤ 0.00 or *p* ≤ 0.000, respectively). Relative expression was calculated, and RPLPO was used as a constitutive control. The data are normalized to 1 using normal tissue as basal expression.

From in silico analysis, several unknown tags overexpressed in HSIL libraries compared with normal cervical libraries were found. Then, the Blast tool was used to identify the genes for these tags and, following these steps, the E was identified. Afterward, TMPO's expression was assessed from CC samples' microarray data, where TMPO's expression was increased in 74% of the samples (Figure 1(a)).

### 3.3. TMPO mRNA Expression: Relative Expression and Isoform Analysis

**Figure 2:**
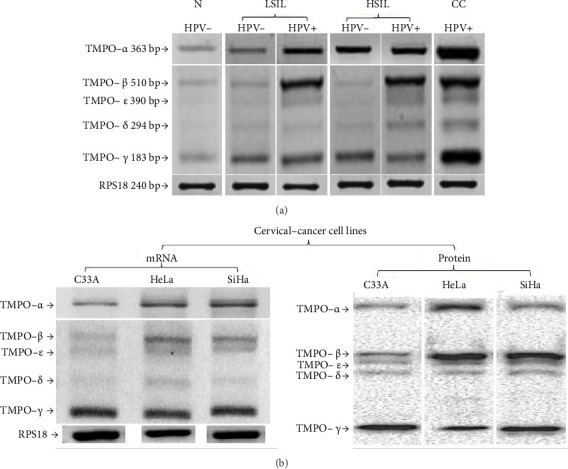
TMPO isoforms at mRNA level detection in cervical tissues. (a) It shows the expression of five of the six TMPO isoforms at the mRNA level by endpoint RT-PCR in different cervical tissues, including LSIL, HSIL, and CC samples. A differential expression is observed in five TMPO isoforms in the samples with or without HPV infection but an increased signal is observed when HPV is positive. The agarose gel electrophoresis is a representative result. (b) Lower panel, as in (a), five TMPO isoforms are expressed in the cervical cell lines. PAGE gel is a representative result. The TMPO protein expression shows five isoforms. It is noted that HeLa and SiHa cells (HPV-positive) present a slightly increased expression compared to C33 cells (HPV-negative).

In order to validate the in silico findings, an RT-PCR assay was performed. Normal or healthy tissue samples showed the TMPO expression, and compared with healthy tissue, the TMPO expression for LSIL, HSIL, and CC samples was 2.13-fold change (FC), 1.66 FC, and 3.01 FC, respectively. Statistical analysis exhibited a significant correlation between TMPO expression in LSIL, HSIL, and CC compared to normal tissue (*p* ≤ 0.00–0.000) (Figure 1(b)). Following the study, the differential TMPO isoform expression was evaluated. Cervical tissues were compared with normal cervical tissues (Figure 2). The findings showed that (1) all cervical samples exhibited five of the six TMPO isoforms (TMPO-*α*, -*β*, -*δ*, -*ε*, and -*γ*); (2) TMPO-*α* expression is increased through cervical lesions until CC; (3) LSIL show similar isoform-expression pattern as normal tissues with slightly increased levels of TMPO-*α* and -*γ* isoforms, but, if HPV is present, a clear increased expression of TMPO-*α* and -*β* is observed (approximately 1.5 folds); (4) HSIL have a more readily detectable presence of TMPO-*β*, -*δ*, -*ε* isoforms when HPV is present, and an increase expression for TMPO-*γ* overall; and finally, (5) CC presents an increased expression in all TMPO isoforms (Figure 2(a) and Table 2).

Also, the TMPO mRNA-α, -β, -δ, -ε, and -γ isoforms were observed in all cell lines (Figure 2(b), left side) and protein (Figure 2(b), right side) levels, observing a differential expression of the TMPO-β isoform among cells with or without the presence of HPV. Interestingly, TMPO-γ isoform did not show any change for either RNA or protein level.

Then, TMPO expression was assessed by IF on the HeLa and SiHa (HPV-positive) or C33A (HPV-negative) CC cell lines. As expected, all cell lines (with or without HPV presence) revealed a cytoplasmic immunosignal (red color) showing the presence of *α*-tubulin, but the immunoreaction (green color) was more readily detectable in HeLa and SiHa cells and for cells in mitotic phase (Figure Supporting 1).

### 3.4. TMPO and MCM2 Protein Expression in Cervical Tissue

**Figure 3:**
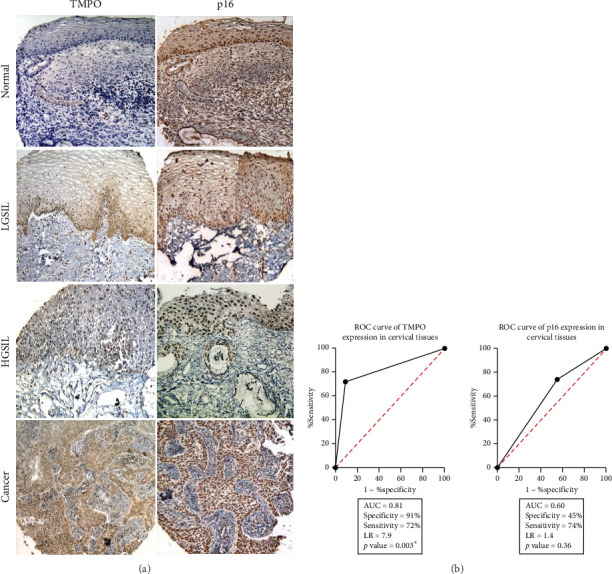
TMPO and MCM2 detection at the protein level in cervical tissues. Immunohistochemistry assay showing in (a) differential TMPO immunostaining or MCM2 immunostaining in normal and cervical lesions. TMPO-negative immunoreaction is observed for the proliferative and differentiated strata level of cervical epithelium, whereas MCM2 is expressed in all tissue. Both TMPO and MCM2 show a similar immunoreaction pattern in LSIL, HSIL, and CC. Brownish nuclear staining is observed for both cases. All tissues were hematoxylin counterstained, X40 original magnification. (b) ROC analysis for TMPO and MCM2 data, where TMPO expression has increased sensitivity (72%) and specificity (91%) in cervical tissues regarding MCM2 results, 74% and 45%, respectively.

Next, it was decided to detect the TMPO at the protein level by IHC on cervical tissues. In normal cervical tissue, we did not observe the TMPO immunosignal (brown color) at any epithelial cell strata. Moreover, a significant difference in TMPO expression (*p*=0.003^∗^) among the LSIL, HSIL, and CC samples was found, in which there were more readily detectable immunosignals. Interestingly, the immunosignal observed was both nuclear and cytoplasmatic (having the latter an unusually strong immunosignal), particularly in both squamous and adenocarcinoma histological CC types (Supporting Figure 2). Again, a significant difference in TMPO expression in cervical cells was found between morphologically normal cells, where null immunoreaction was observed, and abnormal cells, which showed TMPO increased expression (*p*=0.047^∗^) (Figure 3(a)). After the area under the curve (AUC) analysis, the data showed clear differences between TMPO (0.81) and MCM2 (0.60), and the specificity values support these results (Figure 3(b)).

**Figure 4:**
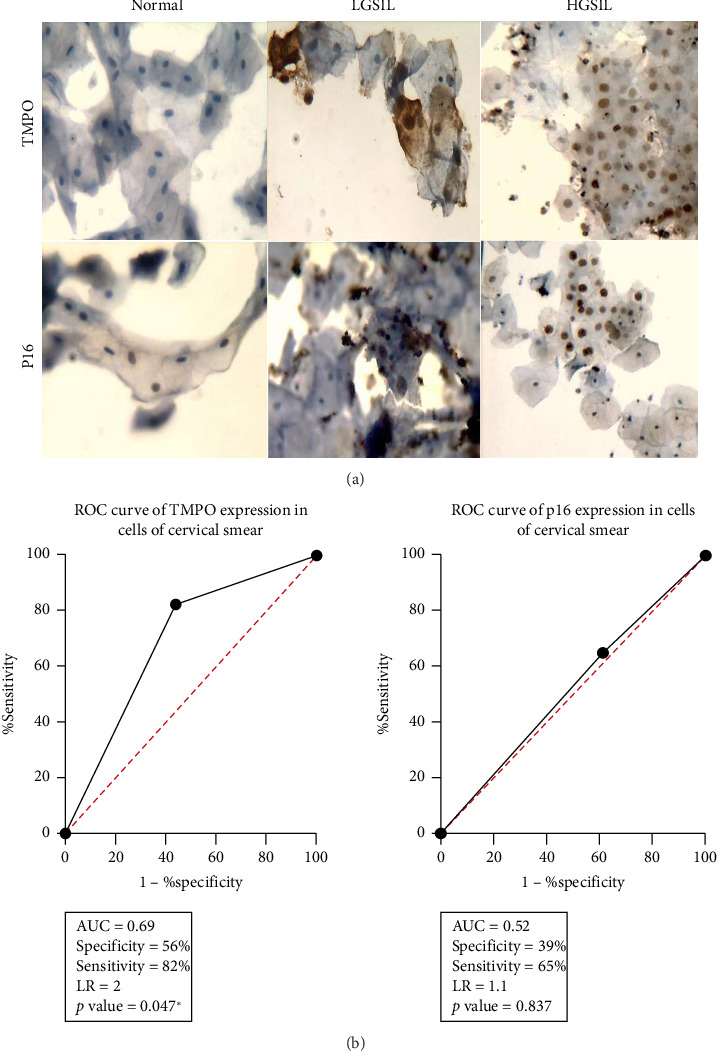
TMPO and MCM2 detection at the protein level in cervical exfoliated cells. Immunostaining assay showing in (a) that a differential TMPO or MCM2 immunostaining in normal and precursor lesions smears is observed. A negative immunostaining for TMPO in normal cells is noted, whereas MCM2 presents a low immunoreaction. Both TMPO and MCM2 show a similar immunoreaction pattern in abnormal cells, where the immunoreaction is more readily detectable. All smears were hematoxylin counterstained, X40 original magnification. (b) ROC analysis for TMPO and MCM2, where TMPO expression has increased sensitivity and specificity in cervical cells regarding MCM2 results.

In addition, the MCM2 and TMPO expression on exfoliated cervical cells were compared by immunohistochemistry. A similar immunoreaction in cells and tissues for LSIL and HSIL was observed; however, again all normal cervical samples were TMPO negative, compared with a MCM2 weak immunoreaction (Figure 4(a)). Furthermore, the sensitivity and specificity of TMPO and MCM2 expression were assessed. The results showed that TMPO had increased sensitivity (82%) and specificity (56%) values in comparison with MCM2, 65% and 39%, respectively, in both tissues (Figure 3(b)) or cells (Figure 4(b)).

## 4. Discussion

In the present work, an increased TMPO isoform expression was observed in the different precursors and invasive cervical tissues.

CC remains a major global health concern suggesting that it is essential to search for reliable molecular markers for its early diagnosis and the need to increase knowledge on the molecular phenomena that are the primary motors of this neoplasia.

It is widely accepted to be the molecular mechanism for cervical carcinogenesis in which E6/E7 oncoproteins interact with their respective p53/pRb targets promoting the epithelial cell proliferation and allowing E2F-dependent gene transcription [21, 22]. It has been demonstrated that some of the responsive genes due to the E2F transcription factor are MCM2 as p16 (known as a surrogate marker of HPV infection) leading to aberrant overexpression in the cervical lesions [22, 23]. Interestingly, the TMPO gene promoter also harbors the E2F DNA-binding motif in its regulatory region [24]. In this sense, these data suggest that p16, MCM2, and TMPO genes share similar mechanisms of molecular regulation via the E2F transcription factor.

As mentioned previously, the *TMPO* can give rise to *α*, *β*, *δ*, *ε*, *γ*, and *ζ* isoforms, indicating that the function of each isoform could be regulated by different alternative splicing mechanisms associated with different cellular processes as immune response [25], organ development [26], malignant transformation [24, 27], and cell cycle [28].

### 4.1. An Increased TMPO RNA Expression in Cervical Lesions

Derived from the in silico analysis of cervical cells, the *TMPO* gene was observed as one of the genes differentially altered in cervical lesions; in this line, it was decided to detect its expression in cervical cells. Our results confirmed an increased TMPO expression in cervical lesions compared to normal tissues. This finding is supported by previous reports where there is evidence that an increase of TMPO expression is observed in transformed cells, indicating that TMPO expression may be one of the molecular mechanisms in human carcinogenesis [24, 27–29].

Then, it was asked if an increase in TMPO RNA expression could correlate to an increase in TMPO protein. The IF assay confirmed that HPV-positive cervical cell lines show an increase in nuclear immunosignal. Furthermore, an intense immunodetection is observed for dividing cells; thus, our results could support the cell cycle's TMPO expression role [24, 25].

### 4.2. Increased Expression of Specific TMPO Isoforms in Cervical Lesions

To know a probable differential expression of the TMPO isoforms, these were evaluated for both RNA and protein. The cervical epithelium cells express five of the six TMPO isoforms (TMPO-α, -β, -δ, -ε, and -γ), and these findings are quite like previously described results for breast tissues [20] and partially in other tumors [24] but significant differences among these were noted.

Our results show that TMPO-*α* has an increased expression in cancer cell lines and cervical tissues, supporting previous reports [20, 24, 27, 29]. However, this expression has been shown for several types of cancer such as breast, larynx, lung, stomach, prostate, and colon cells (for review see 20). The findings presented in the present study as previous reports about the overexpression of TMPO-*α* in cancer could support that E2F is a key contributor to TMPO-*α* increased expression. In this situation, we hypothesize that CC tissues and probably other tissues are susceptible to HPV infection as in oropharynx [20], vagina, and anus, and E7/HPV protein could strongly induce the TMPO-*α* expression. Thus, we could suppose that the TMPO-*α* increased expression could be another probable surrogate marker for HPV infection.

Furthermore, it has been reported that TMPO-α, -β, and -γ isoforms are the most characterized (for review see [20]). These isoforms are constitutively expressed in the cervical epithelial cells more than the other isoforms, but an apparent increase in all isoforms is shown when HPV sequences are present. We also could hypothesize that in HPV infection, all TMPO isoforms are susceptible to increasing their expression and/or increasing the isoforms' stability. All these phenomena could be partially explained because one putative role of the TMPO-*α* isoform is related to cell cycle regulation by binding to the Rb/E2F protein complex, stabilizing it, and transporting it into subnuclear compartments, and/or by association with E2F-dependent promoter genes [30]. The splicing and stability processes for TMPO isoforms could be associated with HPV infection (via HPV/E7), promoting cellular division continually. The uncontrolled cell cycle in infected HPV cells decreases p53 and pRB levels while increasing E2F levels, which in turn permits the overexpression of *E2F*-dependent genes such as the MCM2 marker, among others [4]. Moreover, it has already been described that the TMPO-*β* isoform is capable of modulating E2F transcription activity, altering gene expression [31] and favoring DNA replication [32], and a high rate of cell proliferation [5, 33].

In addition, the TMPO-*γ* isoform is upregulated in differentiated tissues, while the TMPO-*α* and -*β* isoforms are highly expressed in proliferating tissues and cell cultures [31], in part as membrane-chromatin attachment and lamina assembly and may regulate replication by exerting an influence on chromatin structure [34]. These pieces of evidence could strengthen the case for TMPO isoforms to play a key role as potential HPV infection surrogate markers.

### 4.3. Increased Immunostaining of TMPO in Cervical Lesions

To strengthen our findings and to know more about the TMPO protein expression, both cervical samples and cell lines were subjected to the immunodetection assay. TMPO immunodetection was observed only in LSIL, HSIL, and CC samples, and cervical cell lines, suggesting that TMPO expression is implicated in cellular proliferation for probably regulating the cell cycle [5, 20] and protecting the telomeres from damage [30]. Interestingly, in normal samples, TMPO was negative; we hypothesize that it could be due to an extremely low concentration of TMPO isoforms in this cell type. A more readily detectable immunoreaction for TMPO was observed supporting our previous RNA expression data, where there is a direct relationship between RNA and protein. We thought that the presence of viral oncoproteins (HPV infection) would increase splicing mechanisms and/or the half-life of these isoforms.

### 4.4. Detecting TMPO and MCM2 in Cervical Smears

At present, among the promising molecular markers for common cancer are MCMs because their detection identifies those biological processes considered as hallmarks of cancer [35]. Moreover, there is evidence involving MCM2 overexpression in cervical cells because of HPV infection, because MCM2 harbor E2F-responsive elements. In this situation, MCM2 has been considered a molecular marker of HPV infection [36]. In addition, another report indicates that p16/MCM2 dual staining has higher sensitivity than the cytology test and better specificity than the HPV test for SIL lesions, suggesting that p16/MCM2 might be used as an innovative biomarker for CC screening [37].

Taking together the information, the gain of the 3q21 chromosome region (suggesting the gain of MCM2 gene copy number), and its overexpression provoked by E2F transcription factor due to E7/HPV protein presence, could provide a link between HPV infection and the complex molecular event of cervical carcinogenesis. These data and many more strongly support MCM2 testing as an emergent marker for cervical lesions [38, 39]. In order to know about TMPO expression regarding MCM2 expression, IHC and IC assays on cervical tissue or cells from healthy women and with precursor lesions were performed. As expected, our results reveal a similar expression pattern for both proteins. Interestingly, TMPO expression was more sensitive and specific than the MCM2 protein, both in cervical cells and tissues. Thus, if the overexpression of TMPO comprises a common event in cancer, this event would be favored in CC due to the presence of HPV oncoproteins, and more specifically, due to the increasing TMPO-α, -β and -γ isoform levels. In any case, TMPO expression, as well as that of its -α, -β, and -γ isoforms mainly, could represent a promising molecular marker for CC as a MCM2 (or p16) marker.

One weakness in our work is that the mAB utilized recognizes an N-terminal region shared by all TMPO isoforms. Due to the lack of validated TMPO isoform antibodies, we could not demonstrate a specific immunoreaction for each isoform. Furthermore, in the present work, the p16 expression was not included.

In summary, the current results show a robust association between HPV infection and increased expression mainly of TMPO-α, -β, and -γ isoforms in CC cells. Identification of the present data increases our understanding of another molecular pathway of CC cells (in addition to p16), yielding TMPO-α, -β, and -γ isoforms expression evidence, and could be considered as a novel innovative marker for HPV infection diagnosis. Finally, the data suggest a similar contribution of TMPO as MCM2 or p16 does in cervical lesions.

## Figures and Tables

**Table 1:** Correlation of TMPO expression and clinical-pathological variables.

	TMPO-positive	TMPO-negative	*p* value *p* ≤ 0.05	95% CI
Type of tissue				
Normal	0	4		
LSIL	7	1	**0.025**⁣^∗^	0.22–0.28
HSIL	2	1		
CC	13	7		
HPV infection				
Positive	20	7	**0.011**⁣^∗^	0.13–0.18
Negative	2	6		
Alcohol intake				
Positive	5	17	0.662	0.653–0.671
Negative	3	6		
Tobacco				
Smoke	6	5	0.221	0.213–0.229
Nonsmoker	16	4		
Family cancer				
Positive	6	3	0.746	1.00–1.00
Negative	16	6		
Oral contraceptive use				
Negative	3	3	0.327	0.318–0.336
Positive	19	6		
Age				
≤ 45	11	5	0.787	1.00–1.00
> 45	4	11		

*Note:* Bold numbers indicate statistical significance data.

Abbreviations: CC, cervical lesion; HSIL, high-grade squamous intraepithelial lesions; LSIL, low-grade squamous intraepithelial lesions.

⁣^∗^Statistical significance at *p* ≤ 0.05 parameters was associated with TMPO expression by immunohistochemistry.

**Table 2:** Statistical correlation between TMPO isoform expressions and clinical-pathological variables.

	*α*°	*β*°	*ε*°	*δ*°	*γ*°
Normal vs. cervical lesion	0.051 (0.046–0.054)	**0.001**⁣^∗^ (0.000–0.002)	**0.000**⁣^∗^ (0.000–0.000)	**0.000**⁣^∗^ (0.000–0.000)	**0.000**⁣^∗^ (0.000–0.001)
HPV infection	0.214 (0.206–0.222)	**0.041**⁣^∗^ (0.037–0.044)	**0.032**⁣^∗^ (0.029–0.036)	0.083 (0.078–0.088)	0.407 (0.398–0.417)
Age > 45 < 45	0.642 (0.633–0.651)	0.840 (0.832–0.847)	0.854 (0.847–0.861)	0.679 (0.679–0.697)	0.966 (0.966–0.973)
Tobacco smoke	0.401 (0.392–0.411)	0.177 (0.171–0.184)	0.059 (0.54–0.63)	0.076 (0.76–0.87)	0.519 (0.519–0.539)
Alcohol intake	0.678 (0.668–0.687)	0.859 (0.852–866)	0.861 (0.854–0.867)	0.548 (0.538–0.558)	0.298 (0.289–0.307)
Hormonal contraceptives use	0.719 (0.710–0.728)	0.351 (0.358–0.377)	0.262 (0.254–0.271)	0.100 (0.094–0.105)	1.00 (1.00–1.00)
Family cancer	0.455 (0.445–0.465)	0.924 (0.924–0.934)	0.510 (0.510–0.529)	0.432 (0.432–0.452)	0.651 (0.632–0.651)

*Note:* Confidence interval: CI 95%.

⁣^∗^Statistical significance at *p* < 0,05 parameters associated with TMPO isoforms expression; confidence interval: CI 95%. Bold values numbers indicate statistically significance.

°TMPO splicing isoforms (*α*, *β*, *ε*, *δ*, and *γ*).

## Data Availability

Some more raw data can be found in Mauricio Salcedo doi.10.5281/zenodo.11002521.

## Supporting Information

Supporting Information 1 Supporting Figure 1: Immunofluorescent imaging for TMPO expression in cell lines. TMPO detection in the HeLa, SiHa, and C33A cell lines was performed by immunofluorescence assay. The cells were fixed and treated with the specific primary antibody anti-tubulin or anti-TMPO; the system was revealed by using a second antibody red-label or green-label fluorophores, respectively. The nuclei staining was performed by using the DAPI stain (blue). The images were obtained using specific microscopy filters. Nuclei staining for tubulin and TMPO is observed, further showing a strong immunosignal for the process of dividing cells. Green and red images were merged finally. Scale bar 12 *μ*m is also shown.

Supporting Information 2 Supporting Figure 2: TMPO expression in endocervical epithelium. TMPO null immunoreaction is observed in normal endocervix tissue and a strong TMPO immunoreaction (brownish precipitate) is observed in cervical adenocarcinoma tissue. The tissues were hematoxylin counterstained. 40X original amplification.
