# Supplementary figures and images for: Thymopoietin-α, -β, and -γ Isoforms Increased Expression in Cervical Cancer Cells
Source: Can J Infect Dis Med Microbiol. 2025 Apr 9;2025:1668482. doi: 10.1155/cjid/1668482 (PMC12003041; doi:10.1155/cjid/1668482)

Dapi

 $\alpha$ -tubulin

TMPO

Merge

Hela

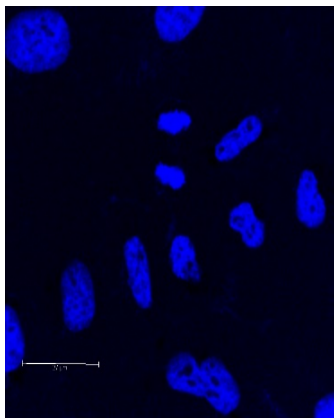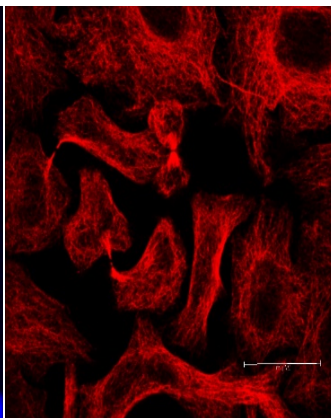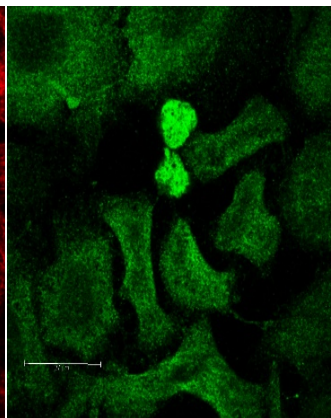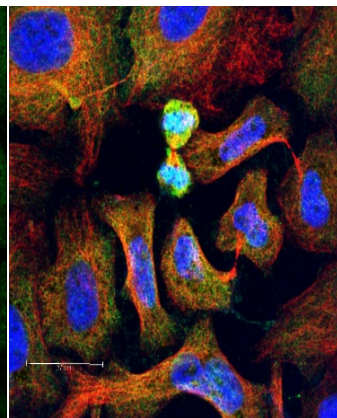

SiHa

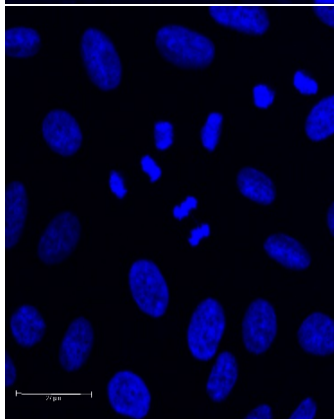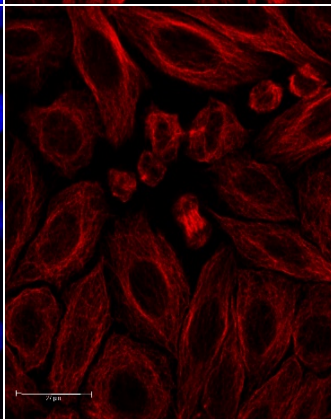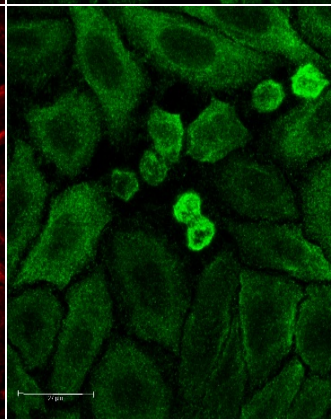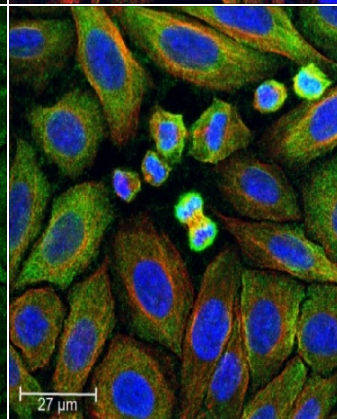

C33A

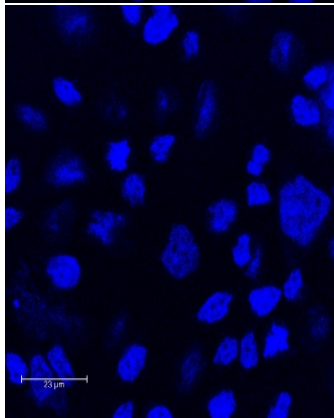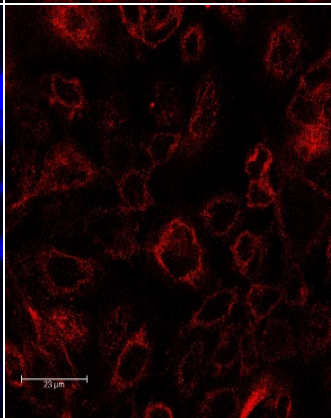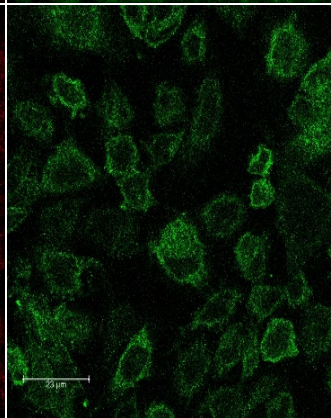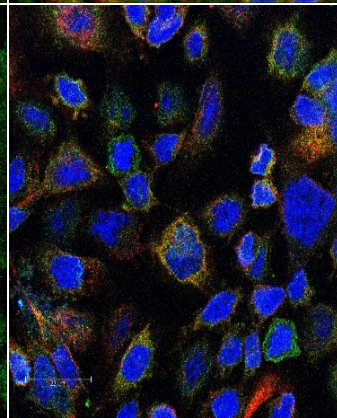

Supplement: Supporting Information 1 — Supporting Figure 1: Immunofluorescent imaging for TMPO expression in cell lines. TMPO detection in the HeLa, SiHa, and C33A cell lines was performed by immunofluorescence assay. The cells were fixed and treated with the specific primary antibody anti-tubulin or anti-TMPO; the system was revealed by using a second antibody red-label or green-label fluorophores, respectively. The nuclei staining was performed by using the DAPI stain (blue). The images were obtained using specific microscopy filters. Nuclei staining for tubulin and TMPO is observed, further showing a strong immunosignal for the process of dividing cells. Green and red images were merged finally. Scale bar 12 μm is also shown. [file 1668482.f1.pdf]

TMPO in normal endocervical epithelium

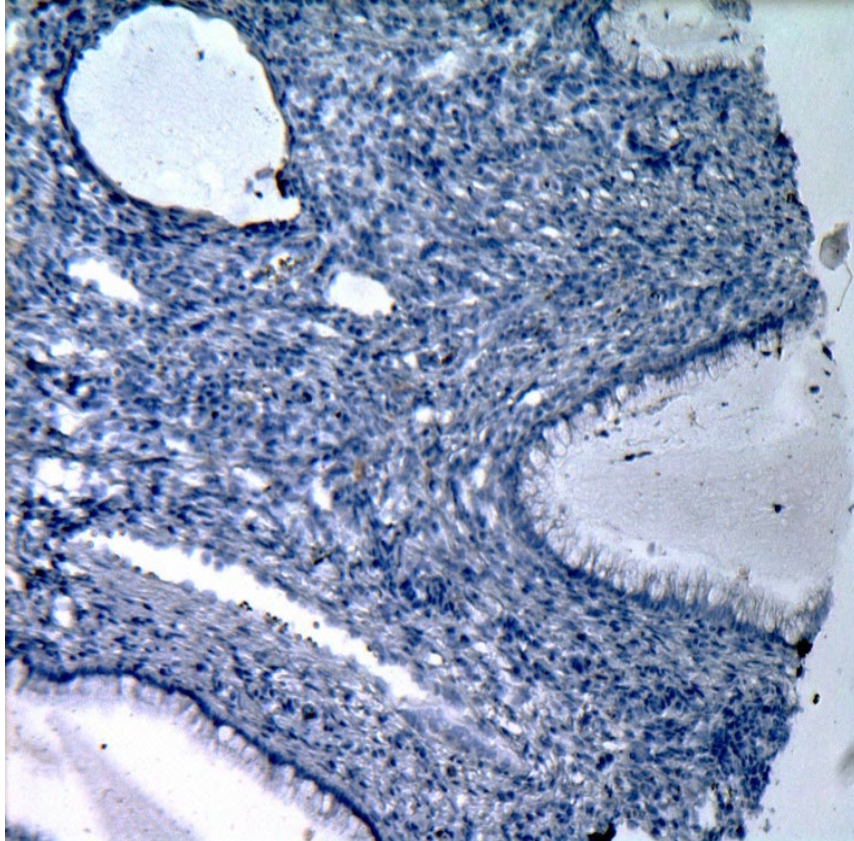

TMPO in cervical adenocarcinoma

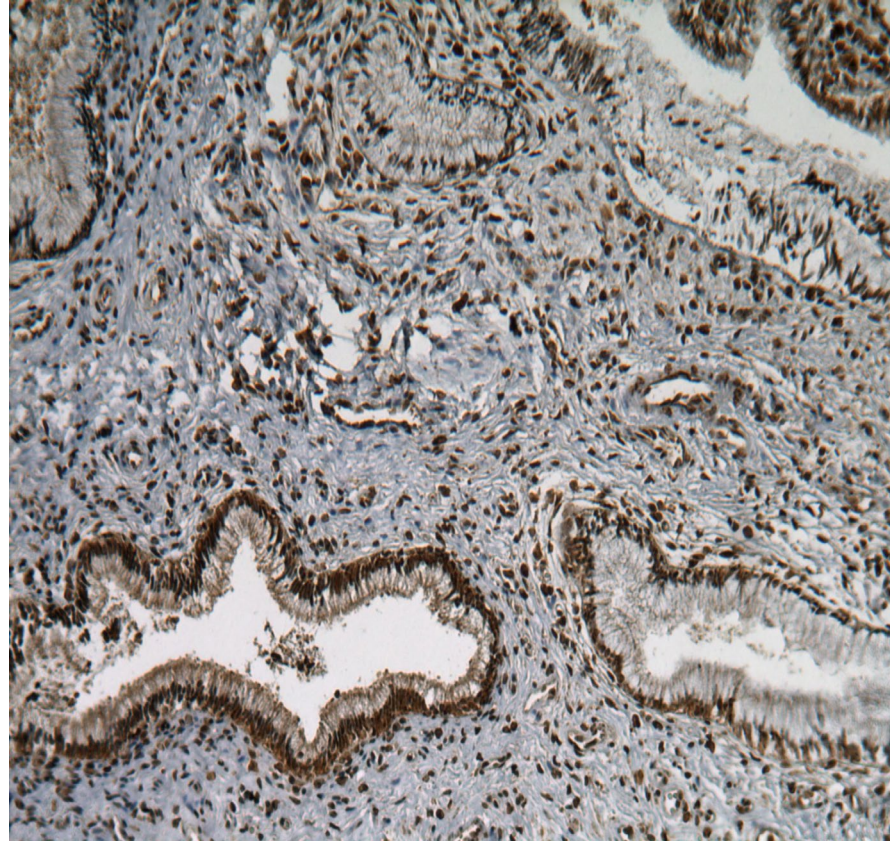

Supplement: Supporting Information 2 — Supporting Figure 2: TMPO expression in endocervical epithelium. TMPO null immunoreaction is observed in normal endocervix tissue and a strong TMPO immunoreaction (brownish precipitate) is observed in cervical adenocarcinoma tissue. The tissues were hematoxylin counterstained. 40X original amplification. [file 1668482.f2.pdf]
